# The Ancient Town Residential Environment of the Elderly in Xiangxi Tujia: Survey, Questions, and Recommendations

**DOI:** 10.3390/ijerph191710820

**Published:** 2022-08-30

**Authors:** Fupeng Zhang, Lei Shi, Simian Liu, Jiaqi Shi, Mengfei Cheng, Tansheng Xiang

**Affiliations:** 1 School of Architecture and Art, Central South University, Changsha 410075, China; 2 Health Building Research Center, Central South University, Changsha 410075, China; 3 College of Architecture, Changsha University of Science & Technology, Changsha 410114, China

**Keywords:** residential environments, indoor air quality, satisfaction, the elderly, ancient town

## Abstract

This study uses behavioral observation, interviews, and questionnaire research to investigate the residential environment. It also evaluates the elderly in four representative ancient towns of Xiangxi, namely, Liye Ancient Town, Furong Ancient Town, Liexi Ancient Town, and Xichehe Ancient Town. It includes indoor air (CO_2_, PM_2.5_, PM_10_) and light intensity monitoring for the residential environment. The results showed that the elderly had a significant sense of frustration and loneliness. Of the elderyly, 70% believed the current living environment had an impact on healthy living, and 45% believed the safety and convenience of the living environment should be improved. More than 80% of the elderly were dissatisfied with their indoor acoustic environment, and more than 70% were dissatisfied with their home transportation. More than 85% of the elderly considered traditional wooden components and spaces to be the source of cultural identity. Furthermore, the average indoor PM_2.5_ concentration during the fire pit fire was 350–600 µg/m^3^, about 4.7–8 times the Chinese standard value. The average concentration of PM_10_ in all rooms was more than 400 µg/m^3^, approximately three times the Chinese standard value. Also, targeted environmental improvement strategies were proposed. The study results provided actual information to develop a systematic approach and a targeted design based on the needs to improve the residential environment of the elderly in ancient cities.

## 1. Introduction

Population aging is one of the major challenges facing most countries worldwide, especially China. China is experiencing a rapid process of population aging, with more than 253 million people aged 60 years or older, accounting for 18.1% population in 2019 [1]. The proportion of the elderly population will predictably reach one-third in 2050 [2]. As the aging population increases, so do China’s challenges to provide adequate and caring living environments for the elderly. It is especially prevalent in the Xiangxi Tujia and Miao Autonomous Prefecture region, where population aging is a serious problem. According to the 7th China Census in 2020, over 470,000 people in these areas are over the age of 65, accounting for China’s 18.99% total population [3]. Physical and psychological disorders in the elderly, such as depression [4], respiratory infections [5], asthma [6], and stroke [7] are affected by the living environment, especially the residential environment. Meanwhile, due to the occurrence of the novel coronavirus SARS-CoV-2, (responsible for COVID-19) in 2019 [8,9], indoor environmental health has become a worldwide concern [10,11], especially for the elderly. Research on suitable and age-friendly living environments is essential for an age-friendly society.

The elderly are more physically and psychologically vulnerable to the effects of their residential environment. There is a great deal of research on the residential environments of the elderly, focusing on their thermal comfort in different regions [12,13], the effects of residential environments [14,15,16], and urban and residential environments and environments with older adults that are friendly to the emotions of the elderly population [17,18,19]. In terms of thermal comfort for the elderly, Zheng et al. [20] developed seasonal and annual adaptive thermal comfort models for the elderly in Xi’an, China, through a subjective questionnaire survey of thermal comfort and objective physical environment tests. Baquero et al. [21] assessed the thermal comfort of elderly people in five Spanish nursing homes in a Mediterranean continental climate during summer using environmental measurements and field surveys. In terms of the impact of the residential environment on the health of the elderly, Li et al. [1]. found significant effects of exterior architectural features, interior spatial layout, home amenities, and indoor environment on depressive symptoms in the elderly through repeated-measure mixed models and the Cox proportional hazards regression models. Abdel-Salam [22] investigated the indoor air quality of residential buildings in Alexandria, Egypt, to assess the daily exposure of elderly people to indoor air pollutants. In terms of the influence and subjective evaluation of the living habits of the elderly on the living environment, Naghibi et al. [23] used a mixed method to study the remaining city spaces to determine the preferences of elderly people, especially their use of open spaces and proposed community gardens for ecological and social interaction. Wang et al. [24] investigated the accessibility and quality of 58 open spaces in an old urban renewal area in Hong Kong. Furthermore, he studied the elderly’s rights to use open urban spaces by questioning their satisfaction with planning and design. Zang et al. [25] examined the effects of residential self-selection and built environment characteristics on the travel behavior of the elderly in Hong Kong through a large-scale public housing program. Thaithatkul et al. [26] conducted a survey and latent class analysis and ordered logistic regression of the elderly in Bangkok, Thailand. It explored the association between subjects’ well-being and the elderly’s mobility, travel behavior, and outdoor activities. Furthermore, sustainability of the living environment for the elderly is one of the main studies. Hu [27] used a qualitative content analysis to review 54 historical research explorations related to the integration of environmental sustainability into the residential environment of the elderly.

According to analysis of the relevant literature, most studies on the residential environment of the elderly have been conducted in urban housing [28,29] and neighborhoods [30,31], and nursing homes [32,33]. Fewer studies relate to the residential environments of elderly people in ancient Chinese towns. However, through analysis, young people in Tujia’s ancient towns are optimistic about the rapid economic development and basic urban public services. It accelerates their willingness to leave and flock to large and medium-sized cities, while the elderly tend to stay back for various reasons like physical function and hometown attachment [34]. Housing construction in ancient Tujia towns also often ignores the need for elderly care. As modern lifestyles change, the aging of traditional dwellings, the elderly’s requirement of special psychological and physiological needs, and the residential environment of ancient towns have afflicted the healthy life of the elderly. Unreasonable planar functions, a lack of accessible care, exposure to highly polluted indoor air, and a lack of space and senior care facilities are some of the challenges. Therefore, it is crucial to study and target their improved residential environment in the ancient towns of western Hunan. We selected representative residences in typical ancient towns to conduct daily behavioral observations, interviews, and questionnaire surveys of the elderly about their residential environment and its indoor air (CO_2_, PM_2.5_, PM_10_) and light intensity monitoring. The residential environment of the elderly there was analyzed and evaluated, and targeted improvement strategies for their care were proposed. The study findings provide useful information for the development of a systematic approach to improving the living conditions of the elderly in ancient towns, as well as for a targeted needs-based design.

## 2. Methodology

### 2.1. Study Area

**Figure 1:**
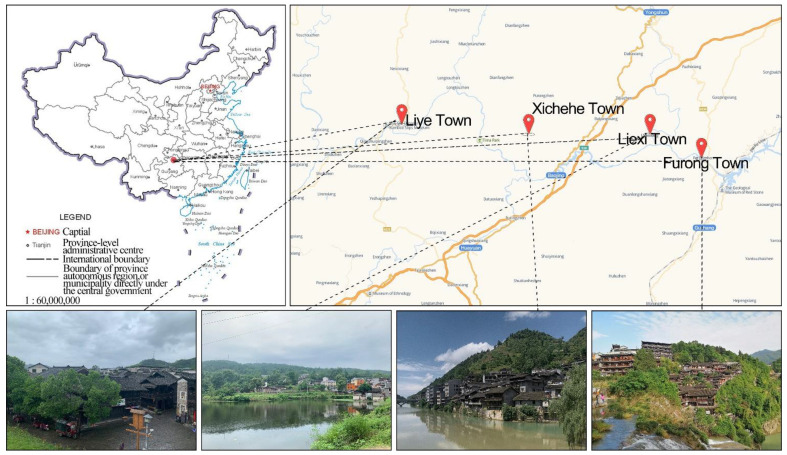
This is a figure. Schemes follow the same formatting.

Xiangxi Tujia and Miao Autonomous Prefecture is the main distribution area of Tujia in China, mainly in the Yongshun, Longshan, Baojing, and Guzhang counties [35]. Western Xiangxi Tujia includes ancient towns with Tujia history, culture, and ethnic traditions dominated by the Tujia people. Ancient towns in western Xiangxi are often built near rivers, facilitating the transport of goods by water while developing commercial trade space on both riverbanks [36]. Based on the ancient Tujia towns’ list published by the Hunan Provincial Department of Construction [37] and the study by Liu [38], this study selected four representative ancient towns, including Liye, Furong, Liexi, and Xichehe, as its study area (Figure 1).

### 2.2. Study Object

**Figure 2:**
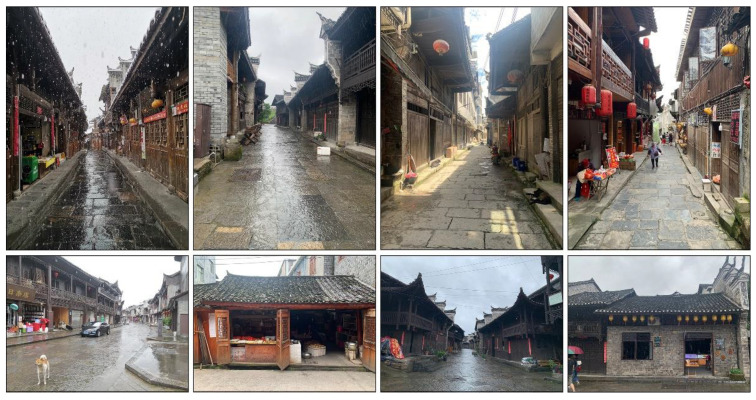
Xiangxi Tujia ancient town houses.

**Figure 3:**
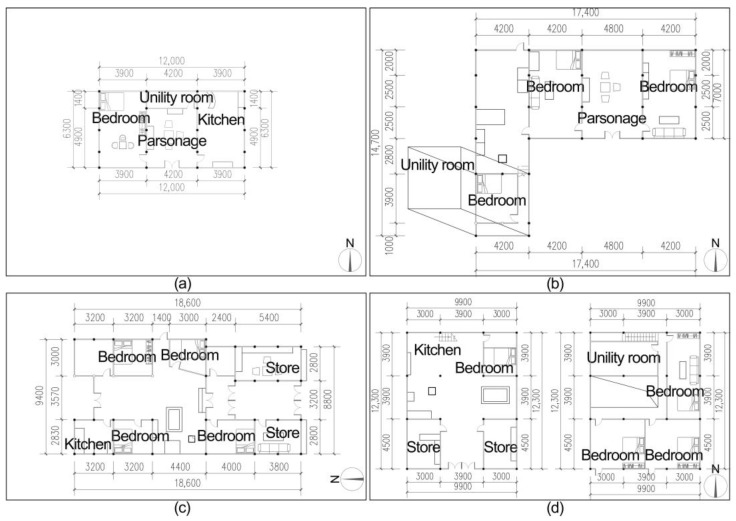
Four representative dwelling planes of the ancient towns in Xiangxi Tujia. (**a**) L-shape dwelling [34], (**b**) Zuozi dwelling [34], (**c**) front store and back house dwelling, (**d**) lower store and upper house dwelling.

Traditional residences in the ancient Tujia town in western Xiangxi, influenced by a unique regional environment, long history and culture, traditional folk beliefs, and other concepts, retain the traditional architectural forms and Tujia lifestyle (Figure 2). The traditional residences there are different from those in the villages. The ancient town dwellings are more complex than the rural ones regarding function, mainly including two categories of three-room and shop-residential dwellings. The three-room dwellings mainly include Zuozi and L-shaped dwellings, while the shop-residential dwellings are divided into two types of dwellings, namely front store and back house dwellings and lower store and upper house dwellings. It depends on the location of the store and living space. The shop-residential dwellings in the ancient town also function as stores. The ratio of openings to depths is also often greater in ancient towns regarding architectural scale, forming a plan layout of large depths and small openings. Based on the preliminary research, this study selected four types of dwellings for the elderly, namely, the Zuozi house and L-shaped dwellings, the front store and back house dwellings, and the lower store and upper house dwellings representing the Tujia ancient towns (Figure 3).

### 2.3. Survey

As physical function and living environments change, the physiological, psychological, and explicit needs of the elderly significantly differ from other age groups. The economic levels and lifestyles between cities and ancient towns also differ from the elderly in urban and ancient towns. This study surveyed the evaluation and needs of the elderly in ancient towns in their existing residential environment, including the three aspects of their activities, physical, and psychological characteristics, and the condition of existing residences.

#### 2.3.1. Behavioral Observation

Observation and data collection helped to understand the social interactions of older adults within the nominated ancient towns. The observation was an effective way to collect information on the activities of the older adults and record them for later analysis [39].

Elderly people’s perceptions of the residential environment in ancient towns are related to their daily activities [40]. This study investigated their daily activities in the ancient Tujia town. Specifically, the research team lived with them there for a month, and the study investigated the daily behavioral activities of the elderly in four-floor plans of traditional three-room and shop-residential dwellings and recorded their daily functional spaces and flows.

#### 2.3.2. Interviews

Interviews were used to explore survey techniques that could guide the evaluation of residential environments for older adults. Interviews were unstructured and used a thematic approach rather than a rigid question structure [39].

(1) Physiological characteristics

As the elderly age, their physical functions and sensory abilities decline [41]. Firstly, in terms of their physical condition, the elderly show a varied decline in body height, metabolic rate, and degree of skeletal spinal curvature. Secondly, these changes in body shape will also influence the scale of indoor space and furniture size the elderly require. As their organs decline, the sensory abilities of the elderly also diminish. This study interviewed 120 elderly people in the ancient town about their physiological characteristics, physical function, and sensory ability, including bodily disease and the ability to act, exercise, and do daily work. The sensory ability interview also included hearing, taste, sight, and touch (Table 1). A comparative analysis of the elderly’s physiological characteristics in the old towns and the city was also conducted based on the study of Wu [42].

(2) Psychological characteristics

As the social economy developed, most young people in ancient towns worked and lived in big cities, denying family companionship to the elderly in the ancient areas of Xiangxi Tujia in western China. Meanwhile, the decline of their physical functions and sensory abilities also causes negative psychological conditions for the elderly. The elderly, in this situation in the Tujia ancient towns, have special needs for a residential environment. Therefore, the research team spent a month living with them there and conducted in-depth interviews and records on the psychological characteristics of 120 elderly people. The interviews included four aspects of loneliness, sense of security, sense of belonging, and frustration that strongly influenced the use of traditional residence functions and compared with the psychological characteristics of the urban elderly (Table 1).

(3) Interviews of the residential environment

We conducted in-depth interviews with the elderly in the ancient Tujia town of western Xiangxi about their residential environment. The interviews were designed based on Zhang et al.’s [43] study about the elderly. It included additional modifications based on their needs in the traditional characteristics of the traditional dwelling of the ancient city. The research discovered that the functional spaces of the ancient town dwellings have nine categories: entrance, hall, store, bedroom, storage room, bathroom, transportation space, fire pit, and patio. The interviews included subjective evaluations of the elderly on the safety, convenience, comfort, identification, privacy, and culture of these spaces of the existing residences (Table 2).

#### 2.3.3. Questionnaire Surveys

A questionnaire study on the residential environment was conducted on the elderly in the ancient Tujia town of western Hunan. The questionnaire was designed based on Ao’s [44] and Liao et al.’s [45] study about the residential environment. It included additional modifications regarding the characteristics of the elderly in the ancient town. The survey involved the psychological needs of the elderly in the residential environment, their subjective feelings about the main function rooms, and their need to improve the existing residential houses (Table 3). A total of 200 questionnaires were distributed, out of which 168 valid questionnaires were returned. Respondents answered the questionnaire anonymously. Data security and privacy were protected and used only for the purposes of this academic research. Ethical approval was granted officially.

### 2.4. Field Measurements

**Figure 4:**
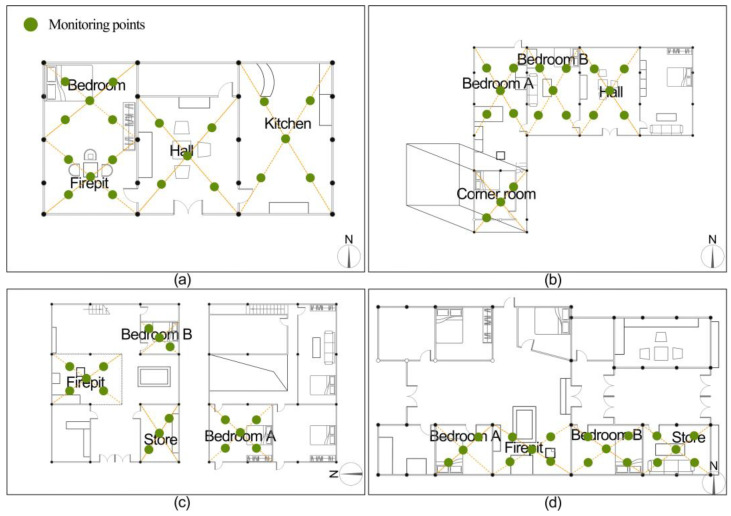
Indoor air quality monitoring points. (**a**) Zuozi dwelling [34], (**b**) L-shape dwelling [34], (**c**) front store and back house dwelling, (**d**) lower store and upper house dwelling.

The monitored dwellings were determined based on the research team’s assessment in the field and discussions with the residents to ensure that these dwellings were representative without affecting the residents’ daily lives. Four representative dwellings in the ancient Tujia town were selected for indoor CO_2_, PM_2.5_, PM_10_, and natural light intensity monitoring (Table 4). The monitoring period was a typical winter day on 26 December 2021. Indoor air monitoring instruments were the AZ-77597 CO_2_ analyzer and the BR-SMART128S air quality instrument (Table A1). According to the Indoor Air Quality Standard [46,47], we set up 5 monitoring points in rooms with an area of 50–100 m^2^, and 3 monitoring points in rooms less than 50 m^2^. The instrument was placed at a measurement site about 1.1 m above the ground and recorded once at an interval of 3 min. The average value of each monitoring point is taken as the room’s air concentration. The indoor light intensity monitoring instrument was the Kurzanleitung testo 540, the monitoring point used the same, and the instrument was 0.75 m above the ground (Figure 4). The selected residential houses did not use air purifiers. Before monitoring, the same sanitary cleaning was done by the residents as usual. During the monitoring process, all south-facing windows and the doors of the hallway and stores were open and residents continued their activities of daily living. A comparative analysis was also conducted based on Chinese indoor air quality standards and light intensity standards for the elderly [48,49] (Table A2 and Table A3).

## 3. Result and Discussion

### 3.1. Behavioral Observation Result

**Figure 5:**
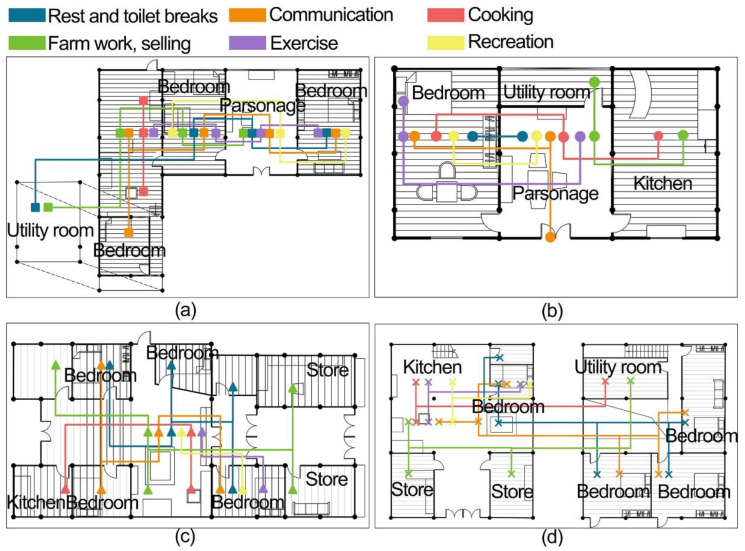
Daily activities of the elderly in the ancient town of Tujia. (**a**) L-shape dwelling, (**b**) Zuozi dwelling, (**c**) front store and back house dwelling, (**d**) lower store and upper house dwelling.

Figure 5 shows the results of the behavioral characteristics of the elderly in the ancient town. The daily activities of the elderly in traditional three-room houses are mainly resting, communicating, cooking, farming, exercising, and entertaining, covering all the rooms. These daily activities in the storehouse-style dwellings are mainly resting and guarding, communicating, cooking, farming, selling, exercising, and entertaining, covering all the rooms. This shows that the elderly use all functions of the existing residence, and that they are indispensable.

**Figure 6:**
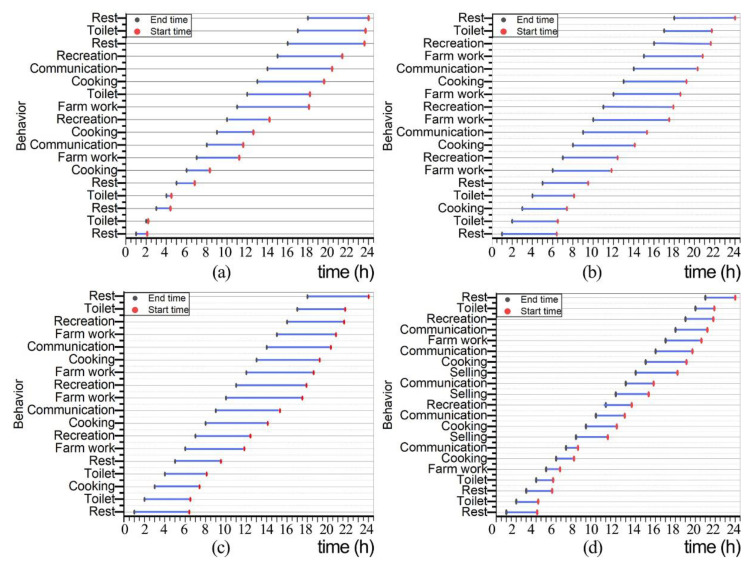
Survey results on the behavioral characteristics of the elderly in ancient towns. (**a**) Zuozi dwelling, (**b**) L-shape dwelling, (**c**) front store and back house dwelling, (**d**) lower store and upper house dwelling.

Figure 6a,b show the activity time statistics of the daily behavior of the elderly in traditional three-room houses. The resting and toileting behavior occupies 42% of their daily routine, which is the longest behavior pattern. It mainly involves the rooms of the hall, fire pit, and bedroom space. Sleeping and resting for the elderly in the ancient town is more regular, but 14.5% of their daily life, including daily cooking and smoking of meat and involved rooms like fire pits and kitchens, with fire pits being used the most. Agricultural activities accounted for 23% of their daily life, including indoor activities such as processing agricultural products, hand-knitting, cutting firewood, cleaning the house, etc. The main rooms involved were the utility room, the hall, the kitchen space, and even miscellaneous items were placed in the bedroom. Communication activities occupy 8% of the daily life of the elderly, involving rooms like the hall, the bedroom, the fire pit, and the threshold. The fireplace and the threshold occupy most of the time. Fitness and recreation occupy 12.5% of their daily life, mainly in the hall and bedroom. Most of them play chess and cards or watch TV with their family members and neighbors in the hall.

Figure 6c,d show the results of the activity time statistics of the daily behaviors of the elderly in the shophouse-style house. As in the traditional three-room residence, resting and toileting behavior still occupies almost half of their daily time. Agricultural work and selling activities occupy 29.58% of their daily time, including selling goods, maintaining and feeding livestock, etc. The main rooms involved are stores, miscellaneous goods, cattle pens, and other rooms. Cooking activities accounted for 13.3% of their daily life, among which the use of fire pits was less than in traditional three-room houses. Communication activities occupy 12.08% of their daily life, and the storehouse-style dwellings are concentrated in the fire pit, bedroom, and patio space centered on the patio. Fitness and recreational activities occupy 7.14% of their daily life, and they usually use the patio and bedroom spaces.

### 3.2. Interviews Results

#### 3.2.1. Physiological Characteristics Interviews Results

**Figure 7:**
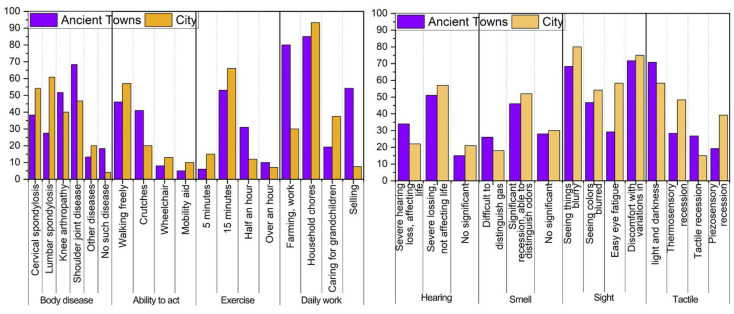
Physiological characteristics and sensory characteristics of the elderly in ancient towns.

Figure 7 shows the results of the physiological characteristics of the elderly in ancient towns. The elderly in ancient towns are less likely to suffer from postural system diseases, except for knee and shoulder diseases. This is because older people in ancient towns have more frequent work and exercise than those in cities. Older people in cities will probably suffer from postural system diseases due to a long-term lack of exercise. In terms of physical activity, the elderly in ancient towns use crutches more frequently than the urban elderly. This is often related to the spatial environment, supporting facilities, and complexity of the indoor space of buildings in ancient towns. In terms of locomotor ability, the elderly in ancient towns are slightly better-off than the elderly in cities. In terms of daily work, old people in old towns work more, many doing plenty of farming and selling activities. Most of the elderly reported that their daily work involved heavier physical activities, such as farming, carrying goods, and raising poultry. This has caused many elderly people in ancient towns to suffer varying degrees of shoulder and knee joint damage and use crutches to move.

Figure 7 shows the results of the sensory characteristics of the elderly in ancient towns. The elderly in the old town and the urban elderly have a significant decline in their auditory function. They have a higher degree of auditory function decline, which affects their lives. In terms of the degree of olfactory function decline, the elderly in ancient towns had a worse olfactory decline. The research also found that due to a profusion of smoke from fire pits, the elderly do not have strong olfactory perception as they have experienced long-term exposure to fire pits for cooking and smoking meat. In terms of vision problems, the elderly in ancient towns performed better regarding eye fatigue, but other aspects, especially changes in light and darkness, produced more acute discomfort. In terms of tactile perception, older people in Guzhen are more sensitive to heat and cold, which may be caused by fewer indoor air conditioners, fans, and other appliances that affect temperature.

#### 3.2.2. Psychological Characteristics Survey Results

**Figure 8:**
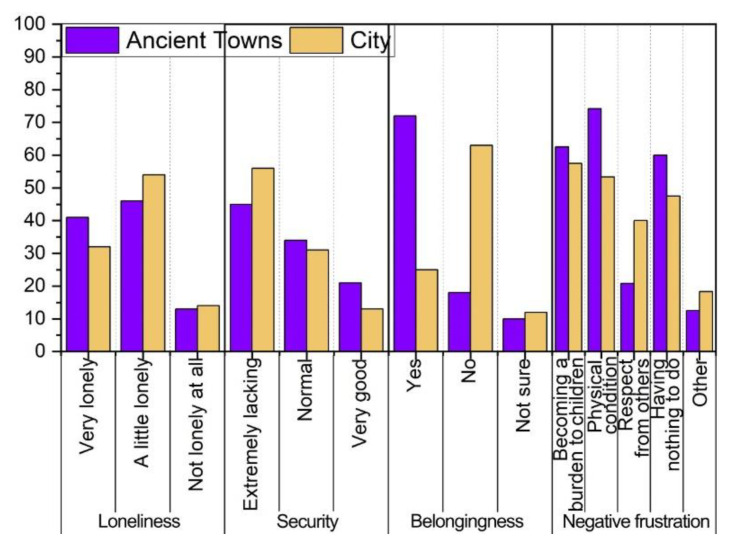
Psychological characteristics of the elderly in ancient towns.

Figure 8 shows the results of the survey on the psychological characteristics of the elderly in ancient towns. They have a stronger sense of loneliness than those in the cities. This is because their children work outside or live in other areas, which results in them living without their family members for a long time. The elderly in ancient towns are also worried and insecure about the safety of their homes and the occurrence of accidents like falls. They have a clearer sense of belonging than the elderly in cities, which is related to the traditional architectural culture and living customs of ancient towns. We see that older people in ancient towns value the respect of others. The fear of increasing their children’s burden and being unemployed due to their physical illnesses cause general negative frustration. It is necessary to conduct adaptive research on the improvement of the built environment to meet the requirements of the unique psychological characteristics of the elderly in ancient towns.

#### 3.2.3. Interviews of Residential Environment Results

**Figure 9:**
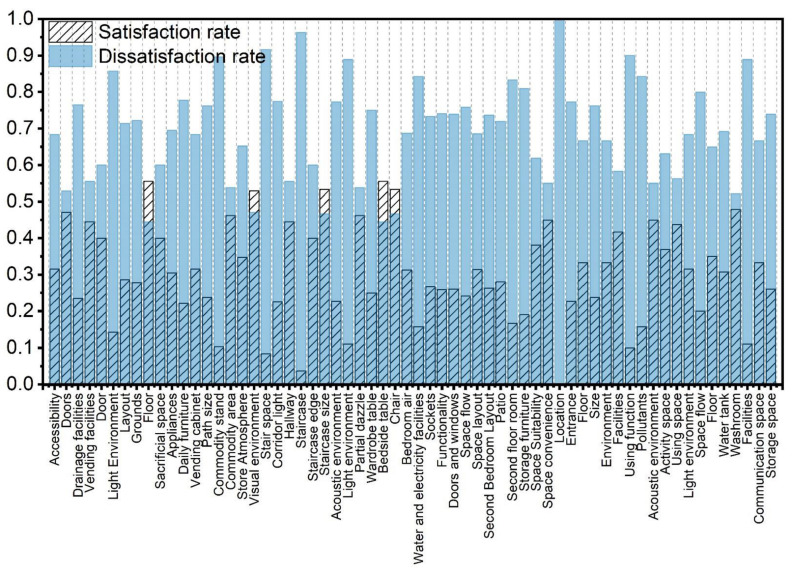
In-depth interviews of the elderly regarding the residential environment.

Figure 9 shows the results of in-depth interviews of the elderly regarding the residential environment. Of seniors, 68% and 76% thought that the threshold and the gutter affected access. Most of the elderly thought that the door and the shop counter on both sides of the entrance affected their daily activities; 70% of seniors thought that the parsonage had insufficient indoor light during the daytime, unreasonable sorting space, and uneven floors. More than half of the seniors thought that the scale and functional distribution of the store was inappropriate. The elderly believed that the staircases are poorly lit, narrow, with no night lighting, poorly scaled steps, missing handrails, etc., affecting their daily behavior and posing serious safety hazards. More than 80% of seniors also believe that stores affect the acoustic environment of the bedroom. They considered the bathroom to be located far away, lacking barrier-free design and safe floor material, and with inadequate consideration of wet and dry separation. Of the seniors, 85% think the fire pit functions improperly, causing indoor air pollution. More than 70% of seniors think the patio houses a variety of daily activities such as cooking, working, negotiating, washing, and storage, but that it has reasonable and inconvenient traffic flow.

We noticed that the elderly in ancient towns believed that the existing residential environment was unsatisfactory. The primary concern of the elderly when proposing improvements for this is the cost. Of the expressed opinions, 45% thought that the safety and convenience of the residential environment need to be improved. This indicates that the safety and convenience of the ancient town dwelling are an important concern for the elderly. The elderly believe that traditional architectural spaces such as fire pits, patios, worship spaces, and water channels provide a sense of cultural belonging. Protection of traditional architectural elements should be considered while improving the existing residential environment.

### 3.3. Questionnaire Surveys Results

**Figure 10:**
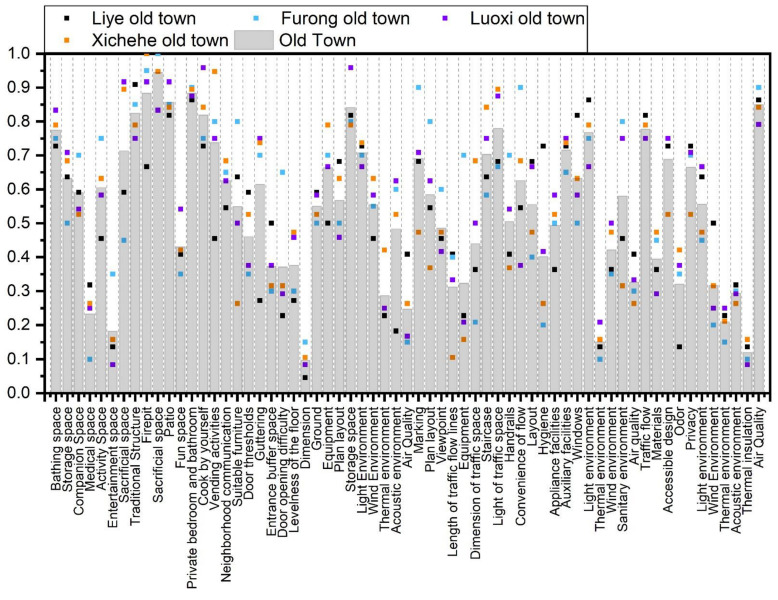
Questionnaire survey on the residential environment of the elderly.

Figure 10 shows the questionnaire survey results on the residential environment of the elderly. In terms of improving quality of life, most elderly people think that there is a lack of bathing, storage, and escort space. Most of the elderly do not require specialized medical space, study room, or theater space. They believe that these functions can be performed in bedrooms and halls, without new specialized rooms. In terms of cultural identity, more than 85% of seniors believe that traditional wooden components, fire pit space, worship space, and patio space are sources of cultural identity in traditional residences. In terms of cultural identity, most of the elderly want their dwellings to provide self-sufficient cooking activities, selling activities, neighborhood communication, and other daily activity behaviors. As for privacy, many elderly people consider the bedroom toilet space in their residential houses problematic, especially the converted toilet and bathing space. As for the entrance in daily life, the elderly are more concerned about the convenience and safety of the entrance, the hall, the bedroom, and other areas where frequent daily activities occur; 70.77%, 76.74%, and 84.94% of them also think that the lighting environment in the hall, the bedroom, and the air environment in the fire pit space of the residential house are uncomfortable. Of the elderly, 58.01% and 55.67% think that the sound environment in the bedroom and the lighting environment in the fire pit have problems, respectively.

### 3.4. Field Measurements Result

#### 3.4.1. CO_2_

**Figure 11:**
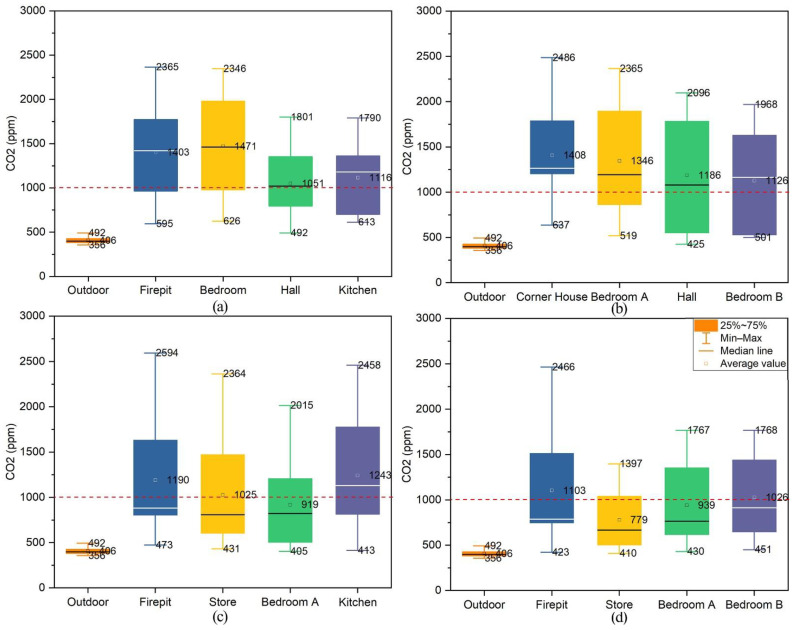
Analysis of CO_2_ concentrations during indoor fires in ancient Tujia town dwellings. (**a**) Zuozi dwelling [34]. (**b**) L-shape dwelling [34]. (**c**) lower store and upper house dwelling. (**d**) front store and back house dwelling.

**Figure A1:**
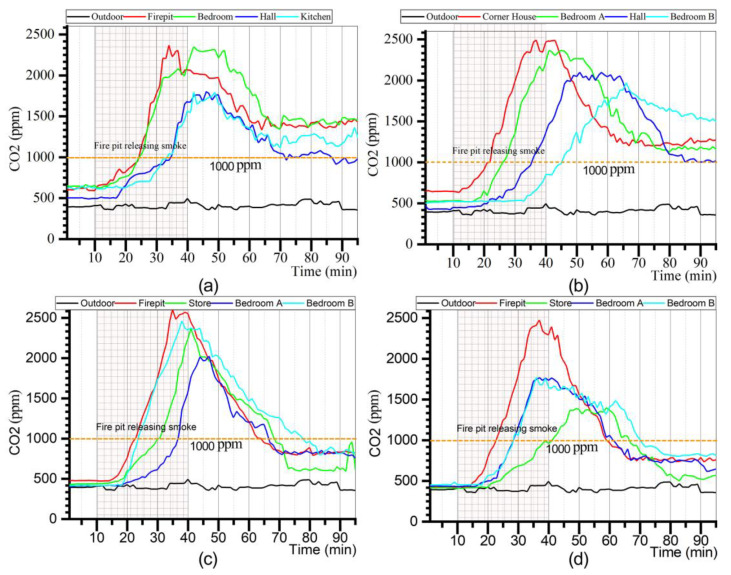
Indoor CO_2_ concentration monitoring results. (**a**) Zuozi dwelling [34], (**b**) L-shape dwelling [34], (**c**) lower store and upper house dwelling, (**d**) front store and back house dwelling.

Figure 11 shows the indoor CO_2_ concentration analysis results of four typical residential dwellings in Xiangxi Tujia ancient town. The maximum values of CO_2_ concentration in the fire pit, bedroom, hall, and kitchen of the Zuozi dwelling were 2365 ppm, 2346 ppm, 1801 ppm, and 1790 ppm, respectively, and the average values were 1403 ppm, 1471 ppm, 1051 ppm, and 1116 ppm, respectively. The maximum values of CO_2_ concentration in the corner house, bedroom A, hall, and bedroom B of the L-shape dwelling were 2486 ppm, 2365 ppm, 2096 ppm, and 1968 ppm, respectively, and the average values were 1408 ppm, 1346 ppm, 1186 ppm, and 1126 ppm, respectively. The maximum values of CO_2_ concentration in the fire pit, store, bedroom A and kitchen of the lower store and upper house dwelling were 2594 ppm, 2364 ppm, 2015 ppm, and 2458 ppm, respectively, and the average values were 1190 ppm, 1025 ppm, 919 ppm, and 1243 ppm, respectively. The maximum values of CO_2_ concentration in the fire pit, store, bedroom A and bedroom B of the front store and the back house dwelling were 2466 ppm, 1397 ppm, 1767 ppm, and 1768 ppm, respectively, and the mean values were 1103 ppm, 779 ppm, 939 ppm, and 1026 ppm, respectively. The monitoring results are displayed in Figure A1.

During the indoor fire period, the average CO_2_ concentrations of the Zuozi dwelling, except for the hall, were much higher than this range. The average indoor CO_2_ concentrations in all the monitored rooms of the L-shape dwelling were higher than the Chinese standard range. Also, the average indoor CO_2_ concentrations in the fire pit and bedroom B of the front store and back house dwelling were higher than this range. The average indoor CO_2_ concentrations in the fire pit and bedroom B of the front store and back house dwelling were above the Chinese standard range, and those of the store and bedroom A were in line with the Chinese standard range. The average CO_2_ concentration in most of the rooms during the fire period in the Xiangxi Tujia dwelling exceeded 1000 ppm. If the residents lived in this environment for a short time, they would feel dull, inattentive, and palpitated, which would harm their health in the long term.

#### 3.4.2. PM_2.5_

**Figure 12:**
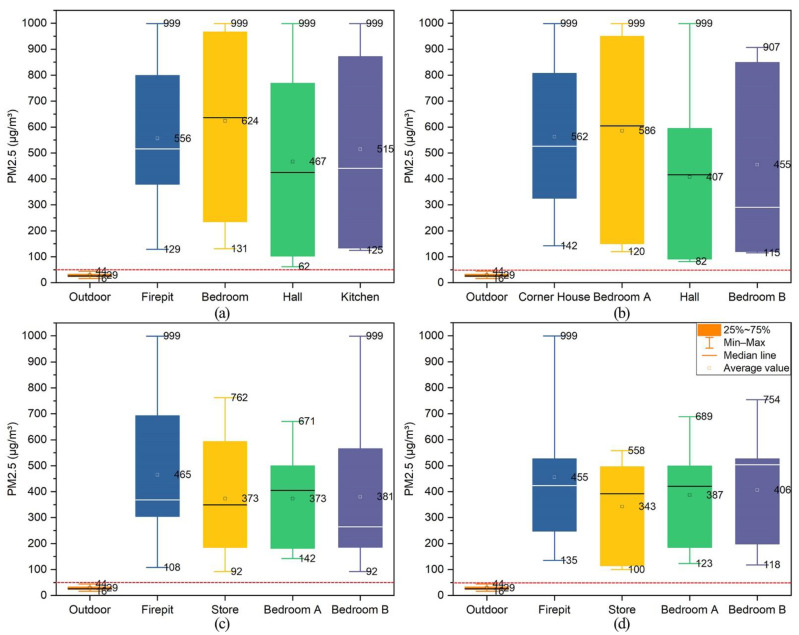
Analysis of PM_2.5_ concentrations during indoor fires in ancient Tujia town dwellings. (**a**) Zuozi dwelling [34]. (**b**) L-shape dwelling [34], (**c**) lower store and upper house dwelling, (**d**) front store and back house dwelling.

**Figure A2:**
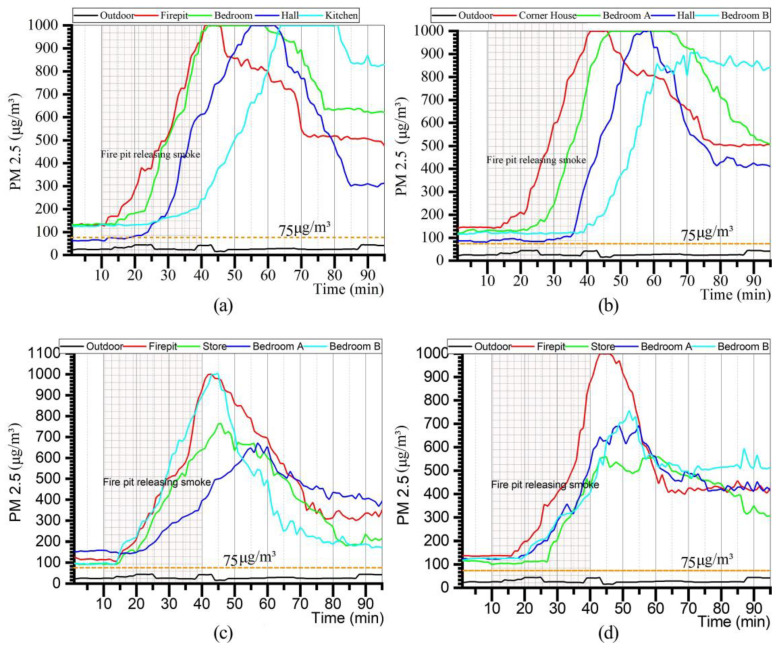
Indoor PM_2.5_ concentration monitoring results. (**a**) Zuozi dwelling [34], (**b**) L-shape dwelling [34], (**c**) lower store and upper house dwelling, (**d**) front store and back house dwelling.

Figure 12 shows the indoor PM_2.5_ concentration analysis results of four typical dwellings in Xiangxi Tujia ancient town. The maximum PM_2.5_ concentrations in all monitored rooms of the Zuozi dwelling were above the instrument’s measurement range (999 µg/m^3^), and the average values in the fire pit, bedroom, hall, and kitchen were 556 µg/m^3^, 624 µg/m^3^, 467 µg/m^3^, 515 µg/m^3^, respectively. Additionally, the maximum concentrations of PM_2.5_ in all monitored rooms of the L-shaped house, except bedroom B, were above the instrument measurement range, and the average values in the corner house, bedroom A, the hall and bedroom B were 562 µg/m^3^, 586 µg/m^3^, 407 µg/m^3^, 455 µg/m^3^, respectively. In addition, the maximum PM_2.5_ concentrations in the fire pit and bedroom B of lower store and upper house dwelling were over 999 µg/m^3^, and the average values in the fire pit, store, bedroom A and bedroom B were 455 µg/m^3^, 343 µg/m^3^, 387 µg/m^3^, 406 µg/m^3^, respectively. The maximum PM_2.5_ concentrations in the fire pit, store, bedroom A and bedroom B of the front store and back house dwelling were over 999 µg/m^3^, 558 µg/m^3^, 689 µg/m^3^, 754 µg/m^3^, respectively, and the average values were 455 µg/m^3^, 343 µg/m^3^, 387 µg/m^3^, 406 µg/m^3^, respectively. The monitoring results are displayed in Figure A2.

The minimum PM_2.5_ concentration (92 µg/m^3^) in all rooms of the four dwellings during indoor fire usage was higher than the Chinese standard values. The average PM_2.5_ concentration in each room ranged from 350–600 µg/m^3^, which was approximately 4.7–8 times higher than the Chinese standard value. If the residents live in this environment for a short time, it will increase the risk of acute respiratory diseases and cardiovascular diseases. Long-term exposure is likely to cause chronic diseases such as lung cancer, chronic obstructive pulmonary disease, and cardiovascular disease. It is extremely dangerous.

#### 3.4.3. PM_10_

**Figure 13:**
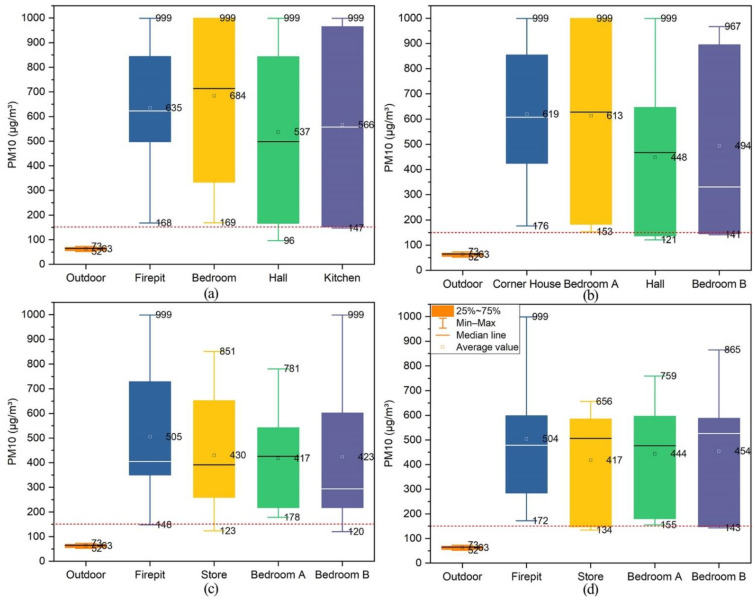
Analysis of PM_10_ concentrations during indoor fires in ancient Tujia town dwellings. (**a**) Zuozi dwelling [34], (**b**) L-shape dwelling [34], (**c**) lower store and upper house dwelling, (**d**) front store and back house dwelling.

**Figure A3:**
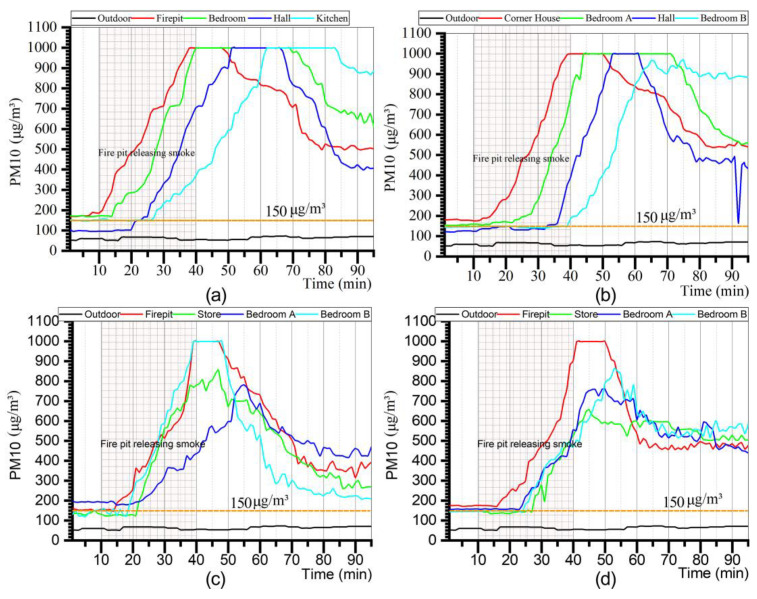
Indoor PM_10_ concentration monitoring results. (**a**) Zuozi dwelling [34], (**b**) L-shape dwelling [34], (**c**) lower store and upper house dwelling, (**d**) front store and back house dwelling.

Figure 13 shows the indoor PM_10_ concentration analysis results of four typical dwellings in Xiangxi Tujia ancient town. The maximum PM_10_ concentrations in all monitored rooms of the Zuozi dwelling were above the instrument’s measurement range (999 µg/m^3^), and the average values in the fire pit, bedroom, hall, and kitchen were 635 µg/m^3^, 684 µg/m^3^, 537 µg/m^3^, 566 µg/m^3^, respectively. The maximum PM_10_ concentrations in all monitored rooms of L-shape dwelling were also exceeded 999 µg/m^3^, and the average values in the corner house, bedroom A, hall, and bedroom B were 619 µg/m^3^, 613 µg/m^3^, 448 µg/m^3^, and 494 µg/m^3^, respectively. Furthermore, the maximum concentrations of PM_10_ in the fire pit and bedroom B of the lower store and the upper house dwelling were greater than 999 µg/m^3^, and the average values in the fire pit, the store, bedroom A and bedroom B were 505 µg/m^3^, 430 µg/m^3^, 417 µg/m^3^, 423 µg/m^3^, respectively. The maximum PM_10_ concentrations in the fire pit, store, bedroom A and bedroom B of the front store and back house dwelling were over 999 µg/m^3^, 656 µg/m^3^, 759 µg/m^3^, 865 µg/m^3^, respectively, and the average values were 504 µg/m^3^, 417 µg/m^3^, 444 µg/m^3^, 454 µg/m^3^, respectively. The monitoring results are displayed in Figure A3.

During the period of indoor fire use, the average concentration of PM_10_ in all rooms of the four dwellings was above the Chinese standard values. The average PM_10_ concentration in each room of the Zuozi dwelling ranged from 537–684 µg/m^3^. The average PM_10_ concentration in each room of the commercial and residential dwellings was similar, ranging from 417–404 µg/m^3^. Long-term exposure to high PM_10_ concentrations can endanger the elderly to diseases. Therefore, we must adopt targeted and effective mitigation strategies.

#### 3.4.4. Light Intensity

**Figure 14:**
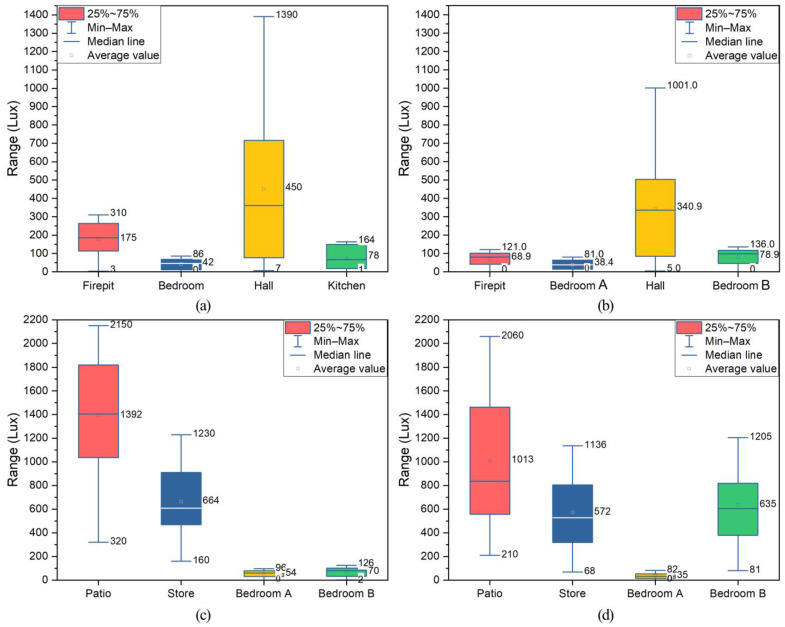
Analysis of light intensity during indoor fires in ancient Tujia town dwellings. (**a**) Zuozi dwelling, (**b**) L-shape dwelling, (**c**) lower store and upper house dwelling, (**d**) front store and back house dwelling.

**Figure A4:**
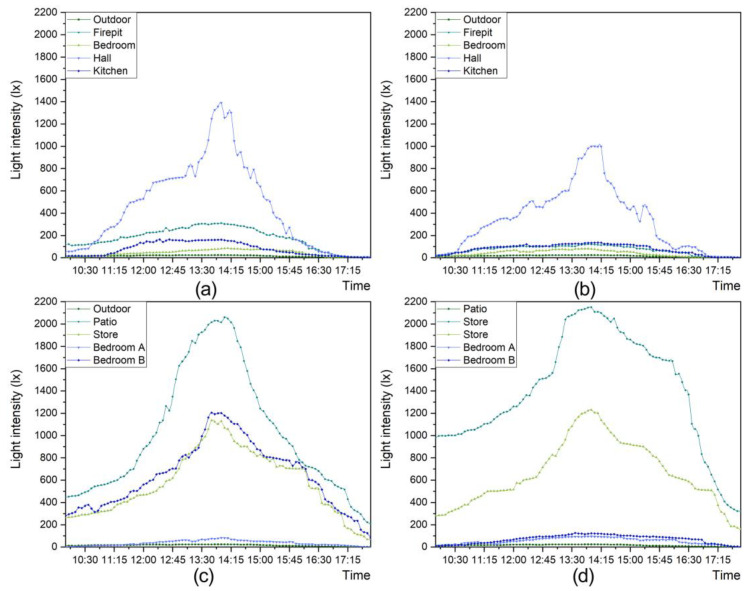
Indoor light intensity monitoring results. (**a**) Zuozi dwelling, (**b**) L-shape dwelling, (**c**) lower store and upper house dwelling, (**d**) front store and back house dwelling.

Figure 14 shows the results of the light intensity analysis of four typical dwellings in Xiangxi Tujia ancient town. The maximum natural light intensity in the fire pit, bedroom, hall, and kitchen of the Zuozi dwelling was 310 lux, 86 lux, 1390 lux, 164 lux, respectively, and the average values were 175 lux, 42 lux, 450 lux, 78 lux, respectively. The maximum natural light intensity in the corner house, bedroom A, hall, and bedroom B of L-shape dwelling was 121 lux, 84 lux, 1001 lux, 136 lux, respectively, and the average values were 68.9 lux, 38.4 lux, 340.9 lux, 78.9 lux, respectively. The maximum natural light intensity in the patio, store, bedroom A and bedroom B of the lower store and upper house dwelling was 2150 lux, 1230 lux, 96 lux, 126 lux, respectively, and the average values were 1392 lux, 664 lux, 54 lux, 70 lux. The maximum natural light intensity in the patio, store, bedroom A, and bedroom B of the front store and back house dwelling was 2060 lux, 1136 lux, 82 lux, 1205 lux, respectively, and the average values were 1013 lux, 572 lux, 35 lux, 635 lux, respectively. The monitoring results are displayed in Figure A4.

The natural light intensity of the hall and fire pit of the Zuo dwelling, the hall of the L-shaped dwelling, the patio, and store of the lower store and upper house dwelling, and the patio and store and bedroom B of the front store and back house dwelling were in line with the range of Chinese standards. In addition to natural lighting, artificial lighting can improve the light intensity of the room. However, our field survey found that artificial lighting could not completely solve the indoor lighting problem of ancient town houses. One of the causes was improper luminaire installation. In some larger rooms, the lamps and lanterns were hung higher than a reasonable height, resulting in insufficient indoor lighting. Moreover, another cause was the luminaire’s lack of power. To save electricity, some elderly people lit up some rooms with light from adjacent rooms. The lack of accessible design and an unsatisfactory indoor lighting environment in the rooms endangers the elderly’s health.

## 4. Problems and Recommendations

### 4.1. The Elderly in the Old Towns Have Multiple Needs

The elderly have different needs for their living environment due to the special economic conditions and the cultural and climatic environment of the ancient town of Tujia. The main six aspects are as follows:

(1) Indoor lighting environment: A good lighting environment can guarantee the elderly’s light requirements and the safety and convenience of various behavioral habits. The research found that the natural lighting environment in ancient towns is poor, and the artificial lighting environment is inconvenient due to economic reasons.
(2) Indoor air quality: The elderly are not sensitive to air pollution, but due to their aging organs, long exposure to highly polluted air can threaten their health.
(3) Residential safety: For indoor stairs, floors, furniture, bathrooms, and other spaces, there is no barrier-free design. The dangers of accidental situations easily threaten the elderly due to their declining physical functions.
(4) Socialization: The elderly in ancient towns are often indoors for a long time when they cannot farm or socialize over long distances. The lack of children’s company also accentuated the strong social needs of the elderly.
(5) Privacy: The elderly often need external help in their lives. The design and construction of traditional Tujia houses do not consider their privacy.
(6) Cultural identity: Several traditional dwellings of the Tujia family have aging problems. Cracking and breakage of traditional architectural wooden components also occurs. The decaying familiar environment of the elderly increases their sense of loneliness and reduces their sense of cultural identity.

### 4.2. Ancient Town Residences Lack Elderly-Friendly Design

The elderly’s special needs were not considered before constructing the dwellings in the Tujia ancient town. As their aging problems increase and the dwellings remain unrenovated, the traditional dwellings are insufficient for the elderly’s healthy living needs. The functions, traffic flow, and the scale of furniture of these dwellings require urgent improvements for the elderly. Traditional dwellings also do not consider the design of accessibility facilities. Indoor air and lighting environments also require improvement.

This study found that the functionality, indoor air quality, and lighting environment of the Tujia dwellings are crucial issues for the residential environment of the elderly. Therefore, targeted recommendations to improve these three areas. Among them, the research found that the source of indoor pollutants in Tujia dwellings was mainly caused by burning activities in the fire pits. Therefore, the recommendations for improving indoor air also included fire pits.

- Floor plan function:

The recommendations include adjusting the layout of the living room, bedroom, kitchen, bathroom, and storage room to make the living environment for the elderly more comfortable and enhance its privacy. For example, the Zuozi dwelling combines the kitchen, dining room, and fireplace to ensure their independence, making the functional flow more independent. The bedroom is separated from the fire pit, and the storage and activity space is increased to increase convenience in the elderly’s daily life.

- Lighting environment:

The research found that the actual lighting area of windows in most residential houses is only half its original window openings and is blocked by indoor furniture or wooden boards. The blockage of windows must be cleared and the size of windows adjusted to improve the indoor lighting environment without destroying its traditional residential style. When adequate light enters the room, the highly reflective ratio finish material can also reflect more light to the depth. This helps improve the traditional dwellings with insufficient light where the depth is large. Interior finishes with higher reflectance ratios can help improve the interior lighting environment. The arrangement of properly powered lights in the room is also an effective method. We need to focus on lighting power and placement to reduce electricity consumption and meet the indoor lighting requirements of the elderly.

- Indoor air quality:

The research found that indoor fire habits in Tujia dwellings are a vital source of pollutants. The usage of fire pits is a cultural practice of the Tujia family, and it cannot be prohibited. It is recommended to adjust the size and position of windows to enhance indoor ventilation to accelerate the diffusion of indoor pollutants to the outdoors. Passive smoke collectors should also be installed above the fire pits so that pollutants thus generated can be discharged indoors.

## 5. Conclusions

This study evaluated the living environment conditions of elderly people in Xiangxi Tujia ancient towns. We conducted interviews and questionnaires in four representative ancient towns, namely, Liye, Furong, Liexi, and Xichehe. We monitored the indoor air quality and natural lighting environment of the representative houses using instruments to identify potential major health problems in the elderly’s living environment. We proposed strategies to improve their living environment in Tujia’s ancient towns while respecting their ethnic beliefs and traditional customs. The conclusions are summarized as follows:

- Behavioral observations showed that the elderly’s daily life in the ancient town included resting, communicating, cooking, farming, fitness, and recreation, involving all functional rooms. The resting and toileting behaviors, farming behaviors, cooking activities, fitness and recreation, and communication activities of the elderly in traditional three-room dwellings accounted for 42%, 23%, 14.5%, 12.5%, and 8% of the elderly’s daily life, respectively. The farming and selling activities, cooking activities, communication activities, and fitness and recreation activities of the elderly in storehouse dwellings accounted for 29.58%, 13.3%, 12.08%, and 7.14% of the elderly’s daily activities, respectively.
- The interview results indicated that the elderly in the ancient town have varying degrees of shoulder and knee joint damage in terms of physiological characteristics and must use crutches to get around. Furthermore, the elderly are less sensitive to indoor air pollution. In terms of psychological traits, the elderly exhibit a strong sense of frustration and loneliness. More than 70% of the elderly believe that the living environment of the hall, bedroom, bathroom, fire pit, and patio has an impact on healthy living; 45% believe that the safety and convenience of their living environment should be improved. While improving the existing living environment, seniors are also concerned about the cost and preservation of traditional elements.
- The results of the questionnaire indicated that 68%, 76%, and 53% of the elderly believe that the threshold, gutter, and stalls on both sides of the entrance have an impact on their daily lives. More than 80% of the elderly were displeased with the indoor sound environment, and more than 70% were displeased with the residence’s traffic. More than 85% of the seniors think that traditional wooden components and space are the sources of cultural identity that the traditional dwellings provide them. The results of the questionnaire match those of the interviews. It should be mentioned that seniors are more concerned with the convenience and safety of the entrance, hall, bedroom, and other areas of frequent daily activities.
- The monitoring results showed that the indoor average CO_2_ concentration and average PM_2.5_ concentration and average PM_10_ concentration, and natural light intensity during the fire activities do not meet the Chinese standard values. The average CO_2_ concentration in more than half of the rooms exceeded 1000 ppm, and the average indoor PM_2.5_ concentration was 350–600 µg/m^3^, about 4.7–8 times the Chinese standard. The average PM_10_ concentration exceeded 400 µg/m^3^, which was roughly 3 times the Chinese standard value. Long-term exposure to highly polluted indoor environments puts the elderly at risk of disease. Effective mitigation strategies are urgently required for the elderly’s indoor air environment in the Xiangxi ancient towns.
- Recommendations for the living environment improvement are proposed to respond to the multiple needs of the elderly and problems of the living environment in the ancient town. This study can provide practical information to systematically improve the living environment of the elderly in ancient towns. It also includes the design of needs-based targeting and a reference to evaluate other types of residential environments for the elderly.

This study has some shortcomings. This study investigated the problems of the residential environment of the Tujia elderly people through detailed interviews and questionnaire research. We used instruments to monitor indoor air pollutants and the natural lighting environment. However, this study only assessed the impact of a single factor on the living environment of the elderly. In fact, there are various factors affecting their residential environment, such as acoustic environment, combined lighting environment with natural and artificial lighting, humid and hot environment, wind environment, and other indoor air pollutants like HCHO, etc., which this study lacks. This study also did not consider the long-term influence and statistical analysis of indoor pollutants and natural lighting environment on the living environment. This study even proposes strategies to improve the living environment for Tujia elderly people that require verification through practical engineering. These will be studied in detail in the future.

## Figures and Tables

**Table 1:** Content of interviews with the elderly in ancient towns.

Basic Features	Comment	Question
Physical Condition	Body disease	Cervical spondylosis, lumbar spondylosis, knee arthropathy, shoulder joint disease, other diseases, no such disease
	Ability to act	Walking freely, crutches, wheelchair, mobility aid
	Exercise	5 min, 15 min, half an hour, over an hour
	Daily work	Farming, work, household chores, caring for grandchildren, selling
Sensory ability	Hearing	Severe hearing loss, affecting life, severe loss, not affecting life, no significant
	Smell	Difficult to distinguish gas, significant recession, able to distinguish odors, no significant
	Sight	Seeing things blurry, seeing colors blurred, easy eye fatigue, discomfort with variations in light and darkness
	Tactile	Thermosensory recession, tactile recession, piezosensory recession, nociceptive recession
Psychological characteristics	Security	Very lonely, a little lonely, not lonely at all
	Loneliness	Extremely lacking, normal, very good
	Belongingness	Yes, no, not sure
	Negative frustration	Becoming a burden to children, physical condition, respect from others, having nothing to do, other

**Table 2:** Content of interviews on the residential environment of the elderly in ancient towns.

Functional Space	Question
Entrance gate	Safety, convenience, satisfaction, size
Hall	Light environment, floor plan, functional rationality, satisfaction
Store	Furniture, functional rationality, environment, size, decoration
Traffic space	Safety and convenience of stairs and corridors
Bedroom	Sound environment, light environment, furniture, plumbing, and electrical facilities, functional layout, indoor traffic, decoration
Storage room	Location, size, convenience, security, furniture
Toilet	Location, decoration, equipment, environment
Fire pit	Safety, pollution situation, functional rationality
Patio	Water tanks, equipment, communication spaces, storage spaces

**Table 3:** Questionnaire surveys the content of the elderly in ancient towns.

Num	Comment	Question
1	Quality of life	Bathing space, storage space, companion space, medical space, activity space, entertainment space, sacrificial space
2	Cultural identity	Traditional structure, fire pit, sacrificial space, patio, fun space
3	Social respect	Private bedroom and bathroom, cook on your own, vending activities, neighborhood communication, suitable furniture.
4	Impact of entrance on daily life	Availability of door thresholds for accessibility, availability of gutters for traffic, size of the entrance buffer space, door opening difficulty, levelness of the floor
5	Impact of the hall on daily life	Dimension, ground, equipment, plan layout, storage space
6	Hall satisfaction	Light environment, wind environment, thermal environment, acoustic environment, air quality
7	Stores satisfaction	Recognition of signs, reasonableness of plan layout, openness of view, length of traffic flow lines, ease of operation of equipment
8	Impact of traffic space on daily activities	Dimension of traffic space, the material of stairs, illumination of the traffic space, height and position of handrails, complexity of traffic flow
9	Impact of traffic space on daily activities	Layout, hygiene, ease of operation of household appliances, availability of auxiliary facilities, opening or not of windows
10	Bedroom environment	Light environment, thermal environment, wind environment, sound environment, air quality
11	Toilet	Traffic flow, materials, with or without barrier-free design, odor, privacy
12	Fire pit	Light environment, wind environment, thermal environment, sound environment, thermal insulation performance, air quality

**Table 4:** Situation of monitored residential houses.

Residence Type	Construction Time	Building Material	Living Conditions
Zuozi dwelling	1980s	Wood, green tiles	2 seniors
L-shaped dwelling	1980s	Wood, green tiles	2 seniors, 1 child
lower store and upper house dwelling	1980s	Wood, green tiles	2 seniors, 2 children
front store and back house dwelling	1980s	Wood, green tiles	2 seniors, 2 children

## Data Availability

Data available on request due to privacy restrictions. The data presented in this study are available on request from the corresponding author. The data are not publicly available due to privacy considerations. Please contact corresponding author before use.

## Appendix A

**Table A1:** Monitoring instrument parameters.

Instrument	Parameter	Accuracy	Measuring Range	Resolution
AZ-77597 Carbon dioxide analyzer	CO_2_	±30 ppm or ±5% (0~5000 ppm)	0~10,000 ppm	1 ppm
BR-SMART128S Air quality instrument	PM_2.5_, PM_10_	±20 µg/m^3^	0~999 µg/m^3^	1 µg/m^3^
Kurzanleitung testo 540	Light intensity	±3 Lux	0~99,999 Lux	1 Lux

**Table A2:** Chinese indoor air quality standards for the elderly.

Pollutant	CO_2_	PM_2.5_	PM_10_
Average value standard	1000 ppm	50 µg/m^3^	150 µg/m^3^

**Table A3:** Chinese indoor light intensity standards for the elderly.

Room	Bedroom	Hall	Kitchen	Living Room
General Activities	100 lux	60 lux	150 lux	150 lux
Fine Activities	200 lux	75 lux	200 lux	300 lux
